# Acute changes to breast milk composition following consumption of high‐fat and high‐sugar meals

**DOI:** 10.1111/mcn.13168

**Published:** 2021-03-03

**Authors:** Ellen Ward, Ni Yang, Beverly S. Muhlhausler, Gabriela E. Leghi, Merryn J. Netting, Matthew J. Elmes, Simon C. Langley‐Evans

**Affiliations:** ^1^ School of Biosciences University of Nottingham, Sutton Bonington Loughborough UK; ^2^ Commonwealth Scientific and Industrial Research Organisation (CSIRO) Adelaide South Australia Australia; ^3^ School of Agriculture, Food and Wine University of Adelaide Adelaide South Australia Australia; ^4^ Women and Kids Theme South Australian Health and Medical Research Institute (SAHMRI) Adelaide South Australia Australia; ^5^ Discipline of Paediatrics University of Adelaide Adelaide South Australia Australia

**Keywords:** breast feeding and maternal nutrition, breast milk, diet, lactation, macronutrients, maternal nutrition

## Abstract

Breast milk composition is influenced by habitual diet, yet little is known about the short‐term effects of changes in maternal diet on breast milk macronutrient concentrations. Our aim was to determine the acute effect of increased consumption of sugar/fat on breast milk protein, lactose and lipids. Exclusively breastfeeding women (*n* = 9) were provided with a control, higher fat (+28 g fat) and higher sugar (+66 g sugar) diet over three separate days at least 1 week apart. Hourly breast milk samples were collected concurrently for the analysis of triglycerides, cholesterol, protein, and lactose concentrations. Breast milk triglycerides increased significantly following both the higher fat and sugar diet with a greater response to the higher sugar compared to control diet (mean differences of 3.05 g/dL ± 0.39 and 13.8 g/dL ± 0.39 in higher fat and sugar diets, respectively [*P* < 0.001]). Breast milk cholesterol concentrations increased most in response to the higher sugar diet (0.07 g/dL ± 0.005) compared to the control (0.04 g/dL) and the higher fat diet (0.05 g/dL) *P* < 0.005. Breast milk triglyceride and lactose concentrations increased (*P* < 0.001, *P* = 0.006), whereas protein decreased (*p* = 0.05) in response to the higher fat diet compared to the control. Independent of diet, there were significant variations in breast milk composition over the day; triglycerides and cholesterol concentrations were higher at end of day (*P* < 0.001), whereas protein and lactose concentrations peaked at Hour 10 (of 12) (*P* < 0.001). In conclusion, controlled short‐term feeding to increase daily sugar/fat consumption altered breast milk triglycerides, cholesterol, protein and lactose. The variations observed in breast milk protein and lactose across the 12 h period is suggestive of a circadian rhythm.

**Key messages**

- Increasing sugar and fat intake in a controlled short‐term feeding study acutely affected breast milk composition.
- Increasing sugar intake significantly increased breast milk triglycerides and to a greater extent than increasing fat intake.
- Breast milk protein and lactose concentrations exhibited significant changes across the 12 h period independent of dietary intake, suggestive of circadian variation in concentrations.

## 1 INTRODUCTION

Breast milk is a fascinating and incredibly adaptive substance that sustains early human life through its nutritional and immunological composition (Ballard & Morrow, 2013). Human milk composition is highly variable and changes in response to many factors, including but not limited to diet, body mass index (BMI) and age of infant (Bachour et al., 2012; Hsu et al., 2014; Mohammad et al., 2009). Mothers with high BMI have been reported to produce breast milk with a pro‐inflammatory fatty acid profile (Panagos et al., 2016) as well as higher fat and lactose concentrations across lactation according to Leghi, Netting, Middleton, et al. (2020), and higher fat and protein concentrations according to Chang et al. (2015). Breast milk composition also changes across the duration of lactation; protein and calcium levels decrease, whereas lactose concentrations increase as lactation progresses (Hsu et al., 2014). In infancy, there is a high degree of plasticity within developing organs and systems (Hochberg, 2011), and therefore, the nutritional environment a child is exposed to can have lasting effects on physiological and psychological health (Langley‐Evans & Muhlhausler, 2017). Breastfeeding during this period has been associated with a number of health benefits for the infant in comparison to formula feeding, including improved cognitive ability (Evenhouse & Reilly, 2005) and reduced risks of both diabetes and obesity (Owen et al., 2005; Victora et al., 2016).

Research into the influence of habitual maternal diet on breast milk composition shows differing relationships between nutrient consumption and relative concentrations in breast milk. Several studies have suggested that compensatory mechanisms act to maintain adequate concentrations of protein irrespective of maternal nutrition (Lönnerdal, 1986) and amino acid patterns in breast milk are not correlated with amino acids in the maternal diet (Ding et al., 2010). Breast milk protein synthesis is, however, thought to be influenced by maternal amino acid and energy availability in livestock (Bionas et al., 2012). Similarly, breast milk lactose content does not appear to be strongly influenced by changes in dietary intakes in lactating women (Smilowitz et al., 2013). Lactose is known to be synthesised de novo (Anderson et al., 2007), and glucose and galactose are thought to be derived from maternal plasma and generated through hexoneogenesis in human breast tissue (Sunehag et al., 2002). Breast milk fats, however, are derived from a combination of maternal stores, dietary intake and de novo synthesis and appear to be more closely related to maternal dietary intakes (Koletzko, 2016). Trans‐fatty acid and polyunsaturated fatty acid (PUFA) profiles in breast milk have particularly strong correlations with their intakes in the maternal diet (Jonsson et al., 2016; Samur et al., 2009).

Despite a general acceptance of the importance of maternal diet in determining breast milk composition, there is a marked shortage of evidence regarding the short‐term changes in breast milk composition in response to diet. In addition, studies evaluating effects of maternal diet, or relationships between specific dietary components and their levels in breast milk, have typically collected samples at a single time point during the day (Kim et al., 2017) or pooled samples collected over a 24 h period (Aumeistere et al., 2019), rather than taking a time‐course approach. Therefore, to the best of our knowledge, there are no studies investigating the short‐term effects of maternal dietary changes on breast milk composition at multiple time points throughout 1 day. This gap in the evidence base is important as advice for breastfeeding mothers regarding their diet is extremely limited yet could be of huge importance in optimising infants' developmental outcomes.

The aim of this study was to determine the effect of acutely increasing the intake of fat or sugar in the maternal diet on the protein, lactose and lipid concentrations in breast milk over the subsequent 12 h period.

## 2 METHODS

### 2.1 Selection criteria

This study was based on a protocol previously developed by our group (Leghi, Netting, & Muhlhausler, 2020). To be eligible to participate in this study, mothers had to be healthy, without history of diabetes including gestational diabetes, non‐smokers and exclusively breastfeeding a singleton infant born at term and have no major dietary restrictions (e.g., vegan) or allergies (e.g., dairy) and be comfortable with collecting breast milk over multiple time points. Infants needed to be between 6 and 24 weeks of age at the time of enrolment.

### 2.2 Participant recruitment

Mothers (*n* = 10) were recruited for this study using advertisements via social media platforms. Women who expressed interest were offered a screening visit, during which mothers' heights and weights were measured, infants were weighed and questionnaires focusing on health, pregnancy, birth and infants feeding patterns were completed to determine eligibility and for demographic information. A discussion to ensure women had established breast feeding and were solely feeding their infants with breast milk was included as part of the screening visit; women with difficulties breast feeding were not deemed eligible for inclusion. Written‐informed consent was obtained from all mothers during the screening visit, prior to study visits.

### 2.3 Study design

All screening and sample collection sessions took place in participants' homes. All sample collection sessions spanned a full day (~8 a.m. to ~7 p.m.) and were conducted at least 1 week apart.

At Visit 1, participants were supplied with a ‘control’ diet; this was designed with the help of a dietitian to ensure that the diet provided sufficient calories, macronutrients and micronutrients to meet the requirements of breastfeeding women (Department of Health, 1991). On Visit 2, participants received a ‘high sugar’ diet, and on Visit 3, they received a ‘high fat’ diet. The control diet comprised 40% carbohydrate (CHO), 22% protein and 38% fat; by energy, the high‐sugar diet was 50% CHO, 22% protein and 28% fat; and the high‐fat diet was 37% CHO, 18% protein and 45% fat. The high‐sugar diet provided 66 g more sugar and the high‐fat diet 28 g more fat compared to the control diet. Further details of these diets are presented in Table A1. The diets supplied consisted of breakfast, lunch, dinner and two snacks. Breakfast was consumed between 8 and 9 a.m., the specific time breakfast began (e.g., 8.10 a.m.) started the 12 h sample collection period. Lunch and dinner were consumed at 5 and 10 h from breakfast, respectively, and snacks were allowed to be consumed at any time between meals. Women were instructed to consume all food provided. These meals were standardised across participants where possible with exceptions made to accommodate the pescatarian diet (to replace chicken in the evening meal) and some dietary preferences (replacement of peanut butter with a similar spread). Beverages throughout the intervention days were restricted to water and caffeine free tea/coffee. Similar meals and snacks were provided at each of the sessions, with the different fat and sugar content achieved by modifications such as replacement of low‐fat dairy with high‐fat dairy in the breakfast and lunch meals and inclusion of high fat/sugar snacks.

### 2.4 Dietary assessment

Participants were supplied with a 5 day estimated food record diary to complete between the screening visit and Visit 1. This included at least 1 weekend day. Women were instructed to record intake of all beverages, meals and snacks, and brand names were encouraged to be recorded where possible. Questions related to habitual diet including whether women usually trimmed visual fat from meat prior to cooking/consumption, habitual use of low/full fat milks or spreads and cooking habits were also included to help determine overall energy consumption. Dietary data were analysed using Nutritics (Nutrition Analysis Software, Research Edition v5.098) and are presented in Table A2.

### 2.5 Breast milk collection

Participants were asked to express (manually or by breast pump) at least 2 ml of breast milk into sterile 15 ml tubes from either breast. The first sample was expressed before breakfast after an overnight fast (between 8 and 9 a.m.), and this was followed by hourly collections until the last sample, which was collected 1 h after the evening meal. Participants were instructed to collect samples as close to the hourly time frame as possible. Most samples were collected as stand‐alone expressions, and if a sample coincided with feeding, they were collected from the opposite breast whilst the infant was suckling. Samples were immediately frozen in participants' home freezers and transferred on the ice at the end of the day to the laboratory where they were stored at −20°C until initial sample preparation processing and then at −80°C until analysis.

### 2.6 Breast milk analysis

#### 2.6.1 Sample preparation

All samples were defrosted for processing within 1 week of being transported to the laboratory. Processing consisted of centrifugation for 10 min at 3,000 RPM at 4°C. This created a visible ‘cream’ layer at the top of the sample which was discarded. The aqueous component of the sample was pipetted and aliquoted before being frozen at −80°C.

#### 2.6.2 Measurement of protein concentrations

Breast milk sample protein concentrations were determined using a standard Lowry assay protocol (Lowry et al., 1951). Briefly, breast milk samples were diluted and plated in duplicate onto a 96‐well plate. A total of 150 μl of 0.1 M NaOH was added, followed by 50 μl of reagent 1 (2% Na_2_CO_3_, 1% CuSO_4_, 2% KNa tartrate), and the samples allowed to stand for 5 min at room temperature. Reagent 2 (0.1 M NaOH and Folin‐Ciocalteau reagent) was then added, and the samples allowed to stand for a further 20 min at room temperature. Standards were created using bovine serum albumin dissolved in water. Plates were read at 655 nm on a BioRad plate reader.

#### 2.6.3 Measurement of lactose concentrations

Lactose concentrations were determined using liquid chromatography‐mass spectrometry (LCMS). Breast milk samples were diluted in the mobile phase (80% Acetonitrile and 20% H_2_O), then filtered using a syringe filter (0.45 μm, 40 hydrophilic nylon syringe filter, Millipore Corporation) and transferred into sealed glass vials. The standard lactose analysis reported previously (Liu et al., 2019) was used. The LC equipment (1100 Series, Agilent) consisted of a degasser (G1322A, Agilent), a pump (G1312A, Agilent) and an auto‐sampler (G1313A, Agilent). The system was interfaced with a Quattro Ultima mass spectrometer (Micromass, UK Ltd.) fitted with an electrospray ion source. The Luna 5u NH2 100A column (250 × 3.20 mm, 5 μm, Phenomenex) was used to separate lactose at room temperature. Chromatographic separation was conducted using a mobile phase of 80% acetonitrile. The flow rate was set at 0.7 ml/min, and the volume injected was 5 μl. Peaks were determined by comparing retention times to the standard solution made from the lactose monohydrate standard. A calibration curve using a range of six standard solutions (10–150 mg/L of lactose in 80% acetonitrile) was created for quantification of lactose level in the breast milk samples. Samples and standards were run in duplicate or triplicate in a randomised order.

#### 2.6.4 Measurement of triglyceride and cholesterol concentrations

Breast milk samples were diluted for triglyceride (TGL) analysis but not for cholesterol analysis. These assays were completed using Thermofisher Infinity triglycerides or cholesterol kits as per the method reported by Elmes et al. (2011).

### 2.7 Statistical analysis

Results are presented as mean with ± standard error of the mean. Statistical significance was determined using repeated and single measures ANOVA with Tukeys HSD post hoc tests where appropriate, using IBM SPSS Statistics for Windows Version 26. Correlations between the maximal changes in concentrations (i.e., change from concentration at baseline and the hour with peak concentration) of each macronutrient across the 12 h period and maternal habitual diet and maternal BMI were assessed by Pearson's Correlation tests using XLSTAT Version 2019.4.2.63912. A *P* value of <0.05 was considered to be statistically significant.

### 2.8 Ethical consideration

Ethical approval for the completion of this study was obtained from the Faculty of Medicine and Health Sciences Research Ethics Committee: c/o Faculty PVC Office School of Medicine Education Centre B Floor, Medical School Queen's Medical Centre Campus Nottingham University Hospitals Nottingham, NG7 2UH (Ethics reference number:191–1901), and was registered with the Australian New Zealand Clinical Trials Registry (ACTRN 12618000613202).

## 3 RESULTS

### 3.1 Participant characteristics

Of the 10 women who were originally enrolled in this study, nine completed all study appointments and breast milk sampling times. One participant withdrew from the study due to difficulty with sample collection and was excluded from the analyses. The sociodemographic information for the participants and their infants are shown in Table 1. Four mothers were of normal weight, three were overweight and two were obese according to the WHO criteria (World Health Organization, 2021). All mothers were of Caucasian English descent except one who was Caucasian Irish. All mothers were exclusively breastfeeding their infants and had been since birth. Three of the nine mothers were multiparous and had one older child; none were breastfeeding more than one child. The remaining mothers were primiparous. One woman was a pescatarian, whereas the others all consumed meat regularly.

**TABLE 1:** Demographic characteristics of mothers and infants recorded at the start of the study

	Mean	SEM	Range	*N* (%)
Mothers, *n* = 9				
Age (yrs)	33.6	1.1	10.0	
Height (cm)	166.4	2	18.0	
Weight (kg)	73.8	4.7	44.3	
BMI	26.7	1.8	15.3	
Infants, *n* = 9				
Age (wks)	13.9	1.8	16	
Weight (kg)	6.1	0.4	3.6	
Delivery type				
Vaginal				4 (44.4)
Assisted (forceps/ventouse)				2 (22.2)
Caesarean section				3 (33.3)

### 3.2 Dietary analysis

The energy and macronutrient composition of the women's habitual diets, assessed over a 5 day period, is presented in Table A2. There was considerable variation in total energy intake between women (*P* < 0.05), but there were no significant differences in their habitual intakes of carbohydrates, total fat, protein or total sugars.

### 3.3 Effect of higher fat and higher sugar intakes on breast milk composition

**FIGURE 1:**
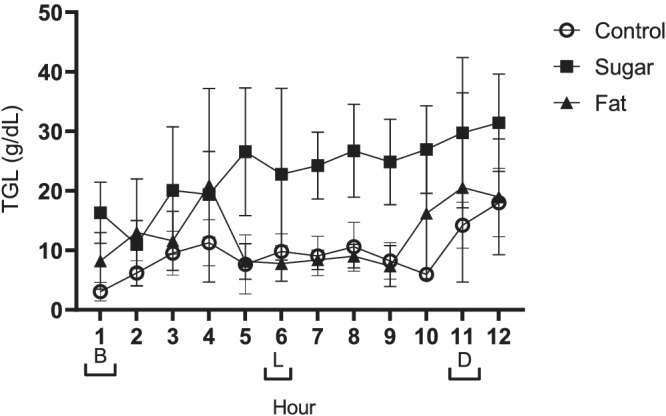
Data presented as mean ± SEM. Meal times are represented on the *x* axis with brackets [B] breakfast, [L] lunch and [D] dinner. Repeated measures ANOVA indicated that milk triglyceride concentration was influenced by diet intervention (fat, sugar or control) (*P* < 0.001), as well as showing a response to time of day (*P* < 0.001; *n* = 9)

Consumption of a diet higher in sugar/fat, compared to the control diet, was associated with significantly greater breast milk TGL concentrations (Figure 1). Consumption of a higher sugar diet was associated with the greatest increase in TGL content, with a mean difference in TGLs of 13.8 g/dL when compared to the control day (*P* < 0.001).

A mean difference in TGLs of 3.05 g/dL was observed between the control and higher fat day (*P* < 0.001). Although breast milk TGLs had increased in response to consumption of the higher fat diet, they were significantly lower when compared to the higher sugar day with a mean difference of 10.71 g/dL (*P* < 0.001).

**FIGURE 2:**
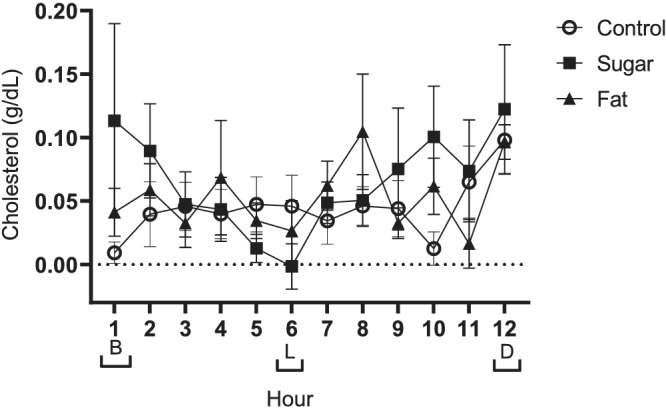
Data presented as mean ± SEM. Meal times are represented on the *x* axis with brackets [B] breakfast, [L] lunch and [D] dinner. Repeated measures ANOVA indicated that milk cholesterol was influenced by increased sugar and fat consumption (*P* < 0.001 and *P* = 0.002, respectively), as well as showing a response to time of day (*P* < 0.001; *n* = 9)

Breast milk cholesterol concentrations increased to a greater extent in response to the higher sugar diet (0.07 g/dL) than both the control (0.04 g/dL) and higher fat diet (0.05 g/dL) (Figure 2) but were not different between the higher fat and control diet (*P* > 0.05).

**FIGURE 3:**
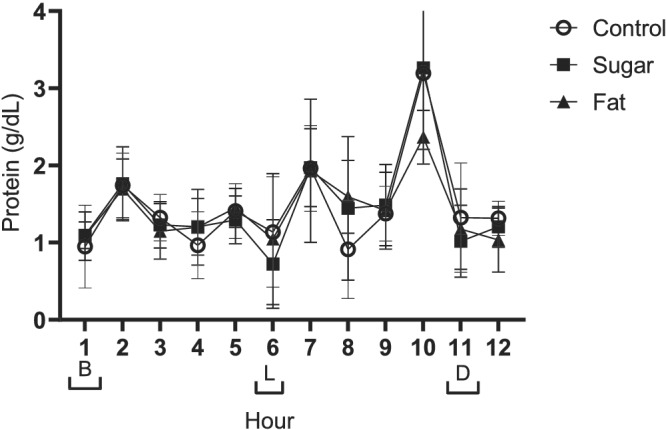
Data presented as mean ± SEM. Meal times are represented on the *x* axis with brackets [B] breakfast, [L] lunch and [D] dinner. Repeated measures ANOVA indicated that milk protein was influenced by increasing fat (*P* = 0.05) as well as showing a strong response to time of day with peaks at Hours 2, 7 and 10 (*P* < 0.001; *n* = 9)

No effect was observed on breast milk protein content in response to consumption of the higher sugar diet, but protein content was lower on the higher fat diet when compared to the control (Figure 3, *P* = 0.05).

**FIGURE 4:**
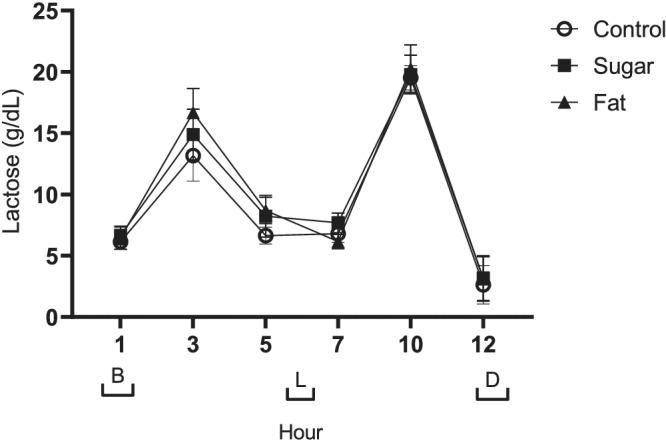
Data presented as mean ± SEM. Meal times are represented on the *x* axis with brackets [B] breakfast, [L] lunch and [D] dinner. Repeated measures ANOVA indicated that milk lactose was influenced by increasing fat (*P* = 0.006), as well as showing a strong response to time of day with peaks at Hours 3 and 10 and a trough at hour 12 (*P* < 0.001; *n* = 9)

Breast milk lactose concentrations were higher following the consumption of the higher fat diet compared to the control diet (*P* = 0.006) but were not affected by the higher sugar diet (Figure 4).

### 3.4 Variations in breast milk composition across the day

There were significant differences in the concentrations of breast milk macronutrients and lipids between collection points across the day, independent of dietary treatment. Concentrations of triglycerides were lower at the start of the day and higher at the end of the day (*P* < 0.001), and concentrations at several individual time‐points across the day were also significantly different (Figure 1).

Breast milk cholesterol concentrations also varied across the course of the day and were significantly higher at the 12 h collection point than at all other time points (*P* < 0.001).

As depicted in Figure 3, breast milk protein concentrations fluctuated across the day and were significantly greater at Hours 2, 7 and 10 than at all other time points (Figure 3, *P* < 0.001).

Lactose concentrations similarly fluctuated across the 12 h collection period, increasing after breakfast and lunch before decreasing towards the end of the sampling period. Breast milk lactose concentrations were lowest at the 12 h time point and highest at the 3 and 10 h time points (Figure 4, *P* < 0.001).

### 3.5 Correlations between change from baseline in breast milk components and habitual maternal intake of macronutrients/maternal BMI

There were no significant relationships between the changes in concentration from the start of the day to the peak concentration across the day in breast milk protein, lactose or cholesterol concentrations and either maternal BMI or habitual dietary intake. On the day the women consumed the control diet, there was a significant negative correlation between women's habitual fat intake and the maximal change in breast milk TGL levels from baseline to end of day (*r* = −0.816, *=*0.048).

## 4 DISCUSSION

The findings of this study suggest that the levels of all macronutrients in breast milk are to some extent responsive to a woman's diet. We have demonstrated that there was a significant increase in breast milk TGLs over 12 h when women consumed a higher sugar diet compared to when they consumed either a control diet or a diet higher in fat. Breast milk cholesterol concentrations were also higher on the day that women consumed the higher sugar diet than on the days when the control or higher fat diets were consumed. In addition, we also observed significant variations in the levels of protein, lactose and lipid concentrations in breast milk over the course of the day, independent of the diet consumed.

The role of maternal diet as a determinant of breast milk composition has been an important research question for decades with the prevailing conclusion being that some breast milk constituents, in particular fatty acids, are influenced by maternal dietary intake (Liu et al., 2019) whereas others (such as protein and lactose) are not (Ding et al., 2010; Smilowitz et al., 2013). The findings of our current study add to these discussions by demonstrating that concentrations of TGLs, cholesterol, protein and lactose are all influenced by relatively small and transient changes in the maternal diet. Specifically, we found that the immediate diet of mothers can influence each of these components in breast milk over the course of the subsequent 12 h.

The responsiveness of breast milk triglycerides to maternal sugar but not fat consumption was unexpected, particularly because breast milk triglycerides have previously been reported to increase in response to a high‐fat diet (Mohammad et al., 2009). However, this discrepency may be due to the fact that the intervention diets in the previous study (Mohammad et al., 2009) were consumed for a longer period (4–7 days) than in our study, providing a much longer period of exposure. A potential explanation for the increased TGL in response to sugar consumption could be that the sugar stimulated an increase in lipogenesis. Parks et al. (2008) completed a feeding study on adults which showed a significant increase in lipogenesis following acute ingestion of fructose with an increase postprandially on lipemia and TGL synthesis. To the best of our knowledge, the changes in breast milk TGL and cholesterol concentrations in response to increased sugar intake have not been reported elsewhere, suggesting a need for further study in the area.

The fluctuations in breast milk protein and lactose concentrations across the 12 h sample collection period, independent of diet, suggest that there is a circadian variation in the concentrations of these factors in breast milk. The variations across the 12 h sampling period reported here challenge the prevailing view that fat concentrations in breast milk are more variable across the day than concentrations of protein and lactose. A study assessing circadian rhythms in breast milk reported no significant difference in protein, fat or carbohydrate throughout the day (Çetinkaya et al., 2017); however, these researchers collected breast milk samples at only 3 points over 1 day, which may not have been frequent enough to detect the patterns that we observed. A further study by Kent et al. (2006) reported that breast milk fat concentrations were higher during the day (10 a.m. to 10 p.m.) than during the night (10 p.m. to 10 a.m.), but again, samples were not collected at regular intervals. Therefore, the differences between our observations and previous studies could simply be due to the sampling strategy we employed (frequent sampling at regular intervals).

Circadian rhythms in breast milk amino acids have been reported in a previous study by Sánchez et al. (2013), in which breast milk samples were collected every 3 h over a 24 h period in colostrum, transitional and mature milk, with particularly substantial variations observed in concentrations of tryptophan, methionine, tyrosine, phenylalanine, glycine and aspartic acid. None of the patterns reported by Sanchez et al., however, correspond to the pattern we observed in total breast milk protein. It is possible that the cumulative effect of the fluctuations in all amino acids and active proteins combined to create the patterns we observed over the 12 h sampling window. Another study also reported a circadian rhythm in breast milk tryptophan concentrations, indicating that these peaked at 3 am and resulted in increased concentrations of the melatonin metabolite (6‐sulfatoxymelatonin) present in infants' urine. The same study reported that breastfeeding was also associated with improvements in sleep parameters when compared to formula feeding, which the authors attributed to the circadian variations in melatonin in breast milk (Cubero et al., 2006).

Although this study adopted a novel approach to investigating the influence of maternal diet on breast milk composition, it is also important to acknowledge its limitations. First, the small sample size makes it difficult to draw robust conclusions, and now that we have proof‐of‐concept of an acute effect of maternal diet on breast milk composition, it will be important to repeat the study in a larger number of women in order to confirm the findings. This study was powered to detect relatively large changes in breast milk composition (40% change in lactose concentration; twofold change in triglycerides); therefore, more subtle changes in breast milk composition across the day or in response to dietary change may not have been detected. The results here are derived from a small group of Caucasian women from one region in England and are not representative of other populations, which may show different responses. Although the frequency of sampling across the day is a strength of the study, we did not collect samples beyond 7 p.m., meaning there could be further compositional changes in response to our interventions and/or diurnal rhythms. Employing a multi‐day intervention could also further support the relationship observed between sugar consumption and breast milk triglycerides and would allow us to determine if habitual diets high in sugar influence breast milk in the longer term as well as acutely. Furthermore, by improving details in future work such as using human milk standards for macronutrient analysis and instructing mothers more stringently on breast milk collection methods (i.e., directing women to collect foremilk only and collecting data on infant feeding times), we could better account for variations resulting from non‐dietary influences. It is important to note that the results presented here will dictate the design of further studies which will aim to clarify and expand on the observations recorded in this study.

In conclusion, this study has shown for the first time that breast milk can be influenced by maternal fat and sugar intake over a period of 12 h. This is an observation that should inform future studies and could be taken into account when discussing diet with breastfeeding mothers. Furthermore, the observations of potential circadian rhythms in breast milk protein and lactose concentrations give insight into breast milk's nutritional composition over a day. These modulations may have an influence on metabolic and hormonal processes which could affect infant development and have lasting effects, a concept which, if further explored, could maximise the benefits of breastfeeding and its influence on infant health.

## CONFLICTS OF INTEREST

The authors declare that they have no conflict of interest.

## CONTRIBUTIONS

EW, BM, ME and SLE designed the research protocol with the inclusion of MN and GB contributions to development of concept. EW collected the data. EW and NY analysed the data. EW, BM, ME and SLE wrote the paper. All authors have read and approved the final manuscript.

## Data Availability

Data from this study will be archived in the University of Nottingham Repository (https://nottingham‐repository.worktribe.com) on the completion of EW's PhD studies and will also be available through the University of Nottingham eTheses database (https://eprints.nottingham.ac.uk/etheses/). Data are also available for analysis on request to the corresponding author.

## APPENDIX A

**TABLE A1:** Breakdown of control diet and intervention diets highlighting the 66.7 g sugar increase and 28.2 g fat increase

	Control (D1)	Higher sugar (D2)	Higher fat (D3)
Energy (kcal)	2,428	2,484	2,597
Carbohydrates (g)	242.6	311.3	240.5
Sugars (g)	83.7	**150.4**	69.3
Fat (g)	101.5	77.5	**129.7**
Saturated fat (g)	35.7	27.5	43.4
Protein (g)	136.1	135.6	117.1

*Note*: The bold signifies the increased amount of relevant macro‐nutrients in the intervention diets.

**TABLE A2:** Habitual dietary intake based on a 5‐day food diary with dietary reference values (DRV) as published by the Department of Health UK, 1991

Participant	Average intake					% of Total energy (TE) intake		
	Energy (kcal)	CHO (g)	Protein (g)	Fat (g)	Sugar (g)	CHO (% TE)	Protein (% TE)	Fat (% TE)
191	2,145	216.9	79.7	94.1	100.9	37.9	14.9	39.5
192	2,410	303.1	91.4	89.9	150.0	47.2	15.2	33.6
193	2,416	261.1	95.8	107.6	82.8	40.5	15.9	40.1
194	2,211	276.6	80.6	83.3	102.5	46.9	14.6	33.9
195	1,893	266.0	66.3	61.1	85.3	52.7	14.0	29.0
196	2,271	258.6	108.5	89.1	130.2	42.7	19.1	35.3
197	1,886	224.13	70.44	73.64	87.54	44.6	14.9	35.1
198	2,099	277.18	69.74	76.61	105.56	49.5	13.3	32.8
199	1,513	177.05	60.87	62.34	115.76	43.9	16.1	37.1
Mean	2,094	251.19	80.37	81.96	106.72			
SEM	91	12.07	4.89	4.76	7.00			
Ref values	2,103–2,175	260	50	70	90	50%	‐	≤35%
